# Assessment of CircRNA Expression Profiles and Potential Functions in Brown Adipogenesis

**DOI:** 10.3389/fgene.2021.769690

**Published:** 2021-10-22

**Authors:** Pengpeng Zhang, Mingxuan Sheng, Chunyu Du, Zhe Chao, Haixia Xu, Xiaofang Cheng, Cencen Li, Yongjie Xu

**Affiliations:** ^1^ Department of Biotechnology, College of Life Sciences, Xinyang Normal University, Xinyang, China; ^2^ Institute of Animal Science and Veterinary Medicine, Hainan Academy of Agricultural Sciences, Haikou, China; ^3^ Institute for Conservation and Utilization of Agro-Bioresources in Dabie Mountains, Xinyang Normal University, Xinyang, China

**Keywords:** circRNA, brown adipocyte, adipogenesis, obesity, high-throughput RNA sequencing

## Abstract

Brown adipose tissue (BAT) is specialized for energy expenditure, thus a better understanding of the regulators influencing BAT development could provide novel strategies to defense obesity. Many protein-coding genes, miRNAs, and lncRNAs have been investigated in BAT development, however, the expression patterns and functions of circRNA in brown adipogenesis have not been reported yet. This study determined the circRNA expression profiles across brown adipogenesis (proliferation, early differentiated, and fully differentiated stages) by RNA-seq. We identified 3,869 circRNAs and 36.9% of them were novel. We found the biogenesis of circRNA was significantly related to linear mRNA transcription, meanwhile, almost 70% of circRNAs were generated by alternative back-splicing. Next, we examined the cell-specific and differentiation stage-specific expression of circRNAs. Compared to white adipocytes, nearly 30% of them were specifically expressed in brown adipocytes. Further, time-series expression analysis showed circRNAs were dynamically expressed, and 117 differential expression circRNAs (DECs) in brown adipogenesis were identified, with 77 upregulated and 40 downregulated. Experimental validation showed the identified circRNAs could be successfully amplified and the expression levels detected by RNA-seq were reliable. For the potential functions of the circRNAs, GO analysis suggested that the decreased circRNAs were enriched in cell proliferation terms, while the increased circRNAs were enriched in development and thermogenic terms. Bioinformatics predictions showed that DECs contained numerous binding sites of functional miRNAs. More interestingly, most of the circRNAs contained multiple binding sites for the same miRNA, indicating that they may facilitate functions by acting as microRNA sponges. Collectively, we characterized the circRNA expression profiles during brown adipogenesis and provide numerous novel circRNAs candidates for future brown adipogenesis regulating studies.

## Introduction

In recent years, the number of obese people is increasing rapidly and becoming pandemic. As obesity is highly associated with metabolic syndromes, such as diabetes, cardiovascular diseases, and even cancer, it severely threatens public health (Blüher, 2019). Obesity develops when the energy intake is more than the energy expenditure. Human adipose tissue mainly includes white adipose tissue (WAT) and BAT. The WAT, which contains large lipid droplets, is the main place of energy storage, whereas BAT contains plenty of mitochondria and converts chemical energy into heat to maintain body temperature (Betz and Enerbäck, 2018). It had been considered that BAT only exists in infants, however recent reports detected functional BAT in adult humans (Leitner et al., 2017). The BAT activity positively correlates with human metabolic rate and reduces energy storage, thus strategies that enhance BAT development and increase BAT activity are considered as possible approaches to combat obesity (Bhatt et al., 2017).

Adipogenesis is the process through which preadipocytes differentiate into adipocytes. Previous studies have identified numerous protein regulators of brown adipogenesis, e.g. Pparγ, Prdm16, and Pgc1α (Shapira and Seale, 2019). In recent years, a lot of studies have been focusing on noncoding RNAs. It is known that many miRNAs (Gharanei et al., 2020) and lncRNA (Squillaro et al., 2020) regulate brown adipogenesis and thermogenesis. Recently, circRNA emerged as a new type of noncoding RNA. CircRNAs are covalently linked non-coding RNAs with neither 5′ caps nor 3’ polyadenylated tails, thus it is more stable than linear RNAs (Li et al., 2018a). In 1979, circRNA was first observed in Hela cells by electron microscopy (Hsu and Coca-Prados, 1979). At that time, circRNA was considered as rare byproducts or spliced intermediates. However, with the routine use of high-throughput sequencing technology, circRNAs are expressed in various organisms (Memczak et al., 2015). CircRNAs are highly conserved between species and the expression patterns are tissue-specific and developmental stage-specific. Recent work suggests that circRNAs play vital roles in various biological activities, such as normal tissue development, pathological processes, and even disease progression. CircRNAs exert their function in several ways. First, circRNAs can bind miRNAs and inhibit their functions. Second, circRNAs can act as protein sponges to promote protein interaction or directly affect protein functions. Third, circRNAs can bind to RNA Pol II complex and act as cis-regulators of transcription. Fourth, a few circRNAs are reported to encode functional proteins (Zhang et al., 2019a).

Recent work indicated that circRNAs are involved in adipose tissue development and obesity. We previously reported that 3,771 circRNAs were expressed in white adipogenesis (Zhang et al., 2021a). Arcinas et al. detected thousands of circRNAs in mouse and human WAT. They identified circTshz2-1 and circArhgap5-2 were essential for adipogenesis (Arcinas et al., 2019). Liu et al. found circSAMD4A (hsa_circ_0004846) was significantly upregulated in obese people. CircSAMD4A affected pre-adipocytes differentiation by acting as the miR-138-5p sponge and regulated EZH2 expression (Liu et al., 2020). Wang et al. discovered that 9,311 circRNAs were expressed in duck pre-adipocytes and adipocytes. Circ-PLXNA1 could regulate adipogenesis by binding to miR-214 and affecting CTNNB1expression (Wang et al., 2020). Zhang et al. found circNrxn2 could promote WAT browning by binding to miR-103 and enhance FGF10 expression levels (Zhang et al., 2019b). Recently, circRNAs were reported to be differentially expressed during brown to white adipose tissue transformation in goats (Zhang et al., 2021b). However, the global expression patterns and functions of circRNAs in brown adipogenesis have not been reported.

BAT primary stromal vascular fraction (SVF) cells which contain a lot of primary BAT preadipocytes are widely used to study adipogenesis *in vitro* (Shan et al., 2016; Oguri et al., 2020). In the present study, we focus on determining the expression profiles and potential roles of circRNAs in brown adipogenesis. The primary SVF cells were isolated from mice interscapular BAT and circRNAs were detected during adipogenesis by RNA-seq. A great number of novel circRNAs were identified and their expression patterns were characterized. Then, we determined the tissue-specific and differentiation stage-specific circRNAs. We also demonstrated that the differential expression circRNAs (DECs) may potentially be involved in regulating brown adipogenesis by acting as miRNA sponges.

## Materials and Methods

### BAT SVF Cells Culture

Primary BAT SVF cells were isolated from C57BL/6J background mice as described (Shan et al., 2016). Briefly, the interscapular brown fat was dissected from 8-week-old mice. Next, the brown fat was cut to fine pieces and digested with 1.5 mg/ml collagenase type I (catalog number SCR103, Sigma-Aldrich) at 37°C water bath for 50 min. The tissue was vortexed every 10 min. Then, the digestion was filtered through 100-μm and 70-μm cell strainers. The digestion was centrifuged at 400 g for 6 min to enrich SVF cells. The cells were seeded into a cell culture plate with growth medium (DMEM and 10% fetal bovine serum). To induce adipogenic differentiation, the growth medium was supplemented with 2.85 mM recombinant human insulin (catalog number I8830, Solarbio), 0.3 mM dexamethasone (catalog number D8040, Solarbio), and 0.63 mM 3-isobutyl-methylxanthine (catalog number I7018, Sigma-Aldrich). After 96 h, the medium was switched to growth medium, supplemented with 10 nM triiodothyronine (catalog number T6397, Sigma-Aldrich), and 200 nM insulin to induce mature adipocytes. For Oil red O staining, cells were fixed with 10% formaldehyde for 5 min and stained with staining solutions (catalog number G1262, Solarbio) according to the instructions.

### RNA-Sequencing

Total RNA of the BAT adipocytes was collected using Trizol (catalog number 15596026, Thermo Fisher Scientific). The total RNA was treated with Epicentre Ribozero rRNA Removal kit (catalog number RZH1046, Epicentre) and RNase R (catalog number RNR07250, Epicentre) to remove ribosomal RNA and linear RNA. Then, the sequencing libraries were prepared by NEBNext Ultra Directional RNA Library Prep Kit (catalog number E7760S, NEB, USA) following the manufacturer’s instructions. At last, the libraries were sequenced on an illumine platform and 150 bp paired reads were generated.

### Computational Analyses of CircRNA

CircRNAs were detected by CIRI2 as previously described (Zhang et al., 2018). Then the sequence data were mapped to mm9 with BWA-MEM algorithm (Li, 2013). CircRNA candidates mapped by at least two reads in both replicated were kept for subsequent study. Then, the DECs were identified with the likelihood ratio test from R package DESeq2 (version 1.10.1) (Hiraike et al., 2017). The DECs were clustered based on their expression patterns by the degPatterns function from the DEGreport package (Pantano, 2021).

### RT-PCR

The cDNA was obtained by using random primers and a reverse transcription kit (catalog number RR037A, Takara). CircRNA sequences were amplified by divergent primers which were designed by Primer3 (https://primer3.ut.ee/) to cover the back-splicing sites. PCR products were extracted by agarose gel DNA extraction kit (catalog number 9762, Takara). Then the purified DNA was sent to perform Sanger sequencing. Then qPCR was performed to examine the expression levels of circRNAs on a LightCycler 96 system (Roche, Germany) using TB Green Premix Ex II (catalog number RR820A, Takara) according to the instructions. The relative expression levels were normalized to 18S.

### CircRNA Expression Patterns Analysis

The expression patterns of circRNAs were classified by the STEM program with No normalization/add 0 option (Ernst and Bar-Joseph, 2006). GO annotations were obtained from the STEM program with the default option.

### CircRNA-miRNA Networks Construction

The circRNA sequences were obtained from package circPrimer (Zhong et al., 2018)and the potential miRNA binding sites were predicted by miRanda (version 3.3a) (John et al., 2004). To obtain more rigorous prediction results, the energy threshold was set to −7 kcal/mol and the score threshold was set to 150, while the other parameters were set to default values. Then, the circRNA-miRNA interaction networks were constructed by Cytoscape (version 3.8.2) (Shannon et al., 2003).

## Results

### Identification of circRNAs in brown Adipogenesis

**FIGURE 1:**
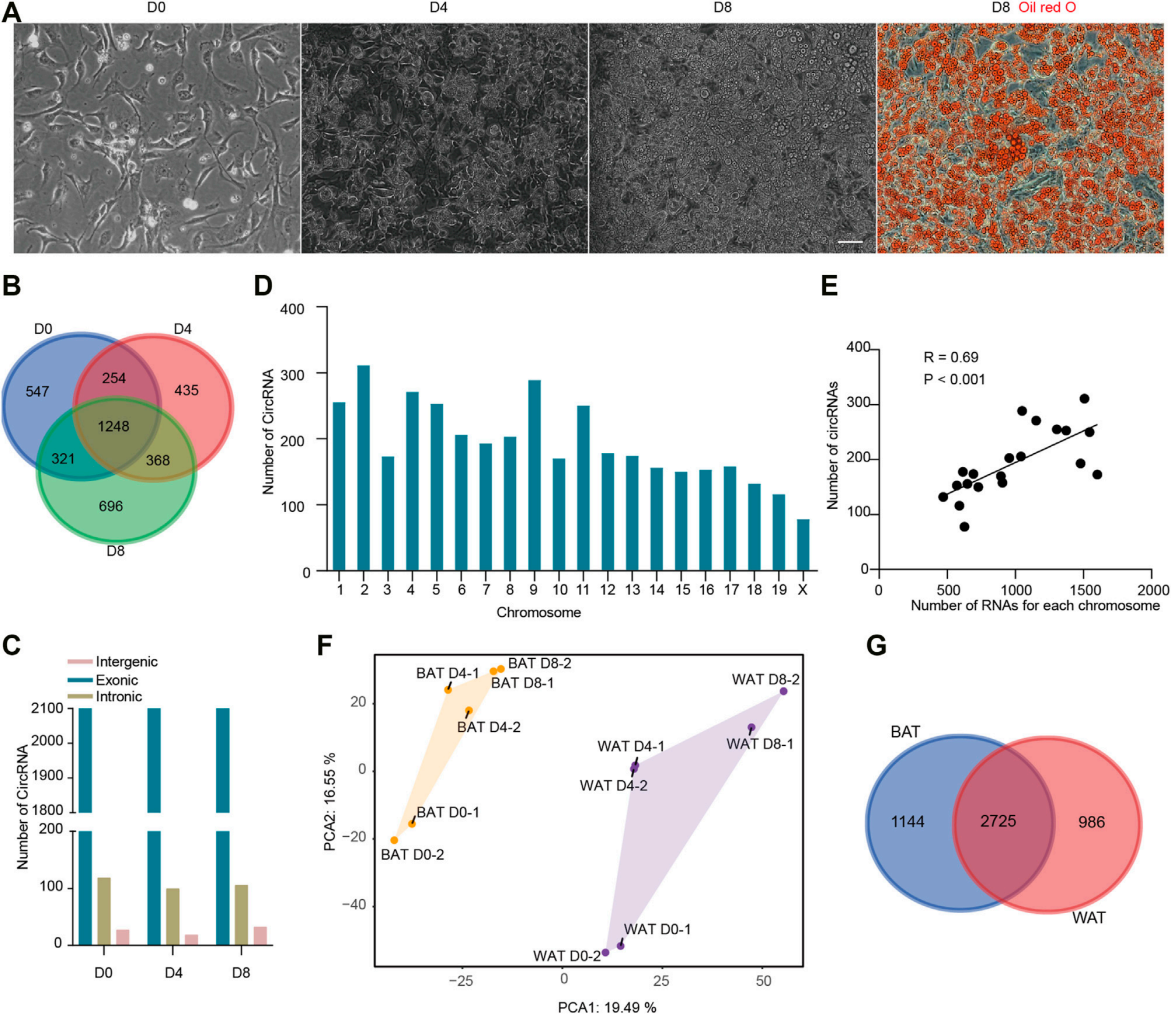
Identification and characterization of circRNAs in brown adipogenesis. **(A)** Representative pictures of BAT pre-adipocytes differentiation. Scale bar = 20 µm. **(B)** The number of CircRNAs identified during BAT pre-adipocytes differentiation. **(C)** The number of circRNA based on back-splicing regions in each adipogenesis stage. **(D)** The number of circRNAs derived from each chromosome. **(E)** Correlation of circRNA and mRNA number from the same chromosome. (F) Principal Component Analysis (PCA) of circRNA expression during BAT and WAT pre-adipocytes differentiation. **(G)** Venn diagrams of circRNAs expressed in BAT and WAT adipogenesis.

To detect circRNAs transcripts in brown adipogenesis, we cultured primary BAT SVF cells and induced them to differentiate. As shown in Figure 1A, the BAT SVF cells were in fibroblast-like spindle shape on day 0 (D0, proliferation stage), whereas the cells round-up on day 4 (D4, early differentiated stage) and were filled with large lipid droplets on day 8 (D8, fully differentiated stage) as indicated by Oil red O staining. Total RNA was collected on D0, D4, and D8 post differentiation. The circRNAs were enriched and then RNA-seq was performed. CIRI2, which is based on back splicing alignment, was used to detect circRNAs. Then the high confident circRNAs with at least 2 reads were detected in both 2 replicates were selected for subsequent analysis. In this way, a total of 3,869 distinct circRNA candidates were detected, indicating that a larger number of circRNAs were expressed in brown adipogenesis (Supplementary Table S1). Notably, up to 36.9% of the circRNAs were novel and not annotated in the circBase database (http://www.circbase.org/). We found that only 32.3% of circRNAs continue to be expressed, while 547, 435, and 696 circRNAs were specifically expressed in proliferation, early differentiated, and fully differentiated stages respectively (Figure 1B). We found that 48 circRNAs were not aligned to the annotated genome sequence, the other 3,821 circRNAs were derived from 2,046 gene locus. Based on our previous study, 20,968 genes were transcribed to mRNA during brown adipogenesis (with a minimum of 4 reads, GEO accession number GSE173710). Thus, as many as 9.61% of genes could produce circRNAs.

According to the back-splicing regions, circRNAs are classified into exonic, intronic, and intergenic circRNAs. We found the ratio of the three types of circRNAs were similar across brown adipogenesis, in which exonic circRNAs were identified as the major type which accounts for more than 93% of all circRNAs, then followed by intronic circRNAs and intergenic circRNAs (Figure 1C). Next, we examined the chromosome distribution of circRNAs. It is noticed that circRNAs were unevenly distributed. Chromosome 2 gave rise to 311 circRNAs which is the greatest, while chromosome X produced only 78 circRNAs (Figure 1D). We suspected that the number of circRNA was related to the length of the chromosome. Thus, we calculated the association between them, and a significant correlation was found (R = 0.51, *p* < 0.05). It is reported that both circRNAs and liner mRNAs are sliced from pre-RNAs, we further examined the association between circRNA and linear mRNA number from the same chromosome (R = 0.69, *p* < 0.001, Figure1E). These results suggested that the biogenesis of circRNA was related to linear mRNA transcription.

As mentioned earlier, BAT and WAT are functionally distinct adipose tissue. To compare the circRNA expression profiles, we collected the WAT SVF circRNA expression data from our previous study (GEO accession number GSE178502), in which the same mice were used as the current study and WAT SVF were isolated from inguinal WAT. Principal component analysis (PCA) analysis showed that samples derived from different tissues located far away, indicating BAT and WAT circRNA expression profiles were largely different (Figure 1F). Then we examined the tissue-specific circRNAs. As shown in (Figure 1G), 2,725 circRNAs were co-expressed in both adipose tissues, while 1,144 and 986 circRNAs were specifically expressed in BAT and WAT, respectively. Thus, almost 30% of the circRNAs were specific to BAT or WAT.

### Alternative Back-Splicing of CircRNAs

**FIGURE 2:**
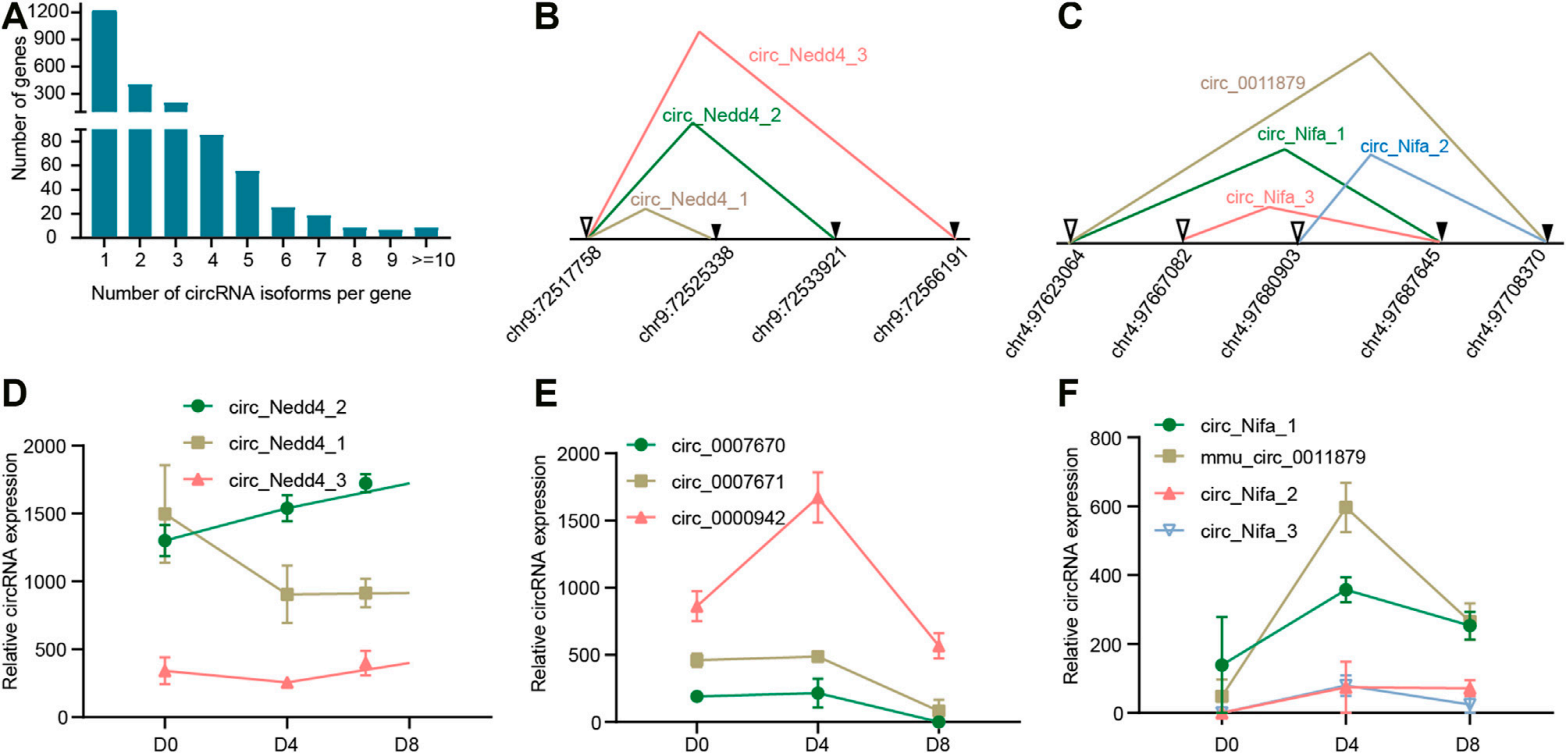
Alternative back-splicing of circular RNAs. **(A)** Distribution of circRNAs variants among genes. **(B)** Generation model of circRNA variants derived from the same acceptor in Nedd4. Splicing acceptor is indicated with ▼, while splicing donor is indicated with▽. **(C)** Generation model of circRNA variants derived from same acceptor or same donor in Nifa. **(D–F)** Expression profile of circRNA isoforms for Nedd4, Glis3, and Nifa across brown adipogenesis.

In most cases, a host gene only produced one circRNA, however, these circRNAs only account for 31.8% of all the circRNAs expressed in BAT. The other circRNAs came from alternative back splicing (Figure 2A). Strikingly, some host genes even produced more than 10 circRNA isoforms, for example, Atrophin 2, which acts as transcriptional co-repressors and plays key roles during embryogenesis (Zoltewicz et al., 2004), produced up to 20 distinct circRNAs. The above results suggest that alternative back-splicing is an important source of circRNA diversity.

As reported, one gene can make several circRNAs by selectively using different splice donors or splice acceptors (Liang et al., 2017). For example, Nedd4 is an E3 ubiquitin ligase that is involved in adipogenesis by regulating Pparγ stability (Yao et al., 2019). Nedd4 could produce three distinct circRNA isoforms by using the same splice acceptor and different splice donors (Figure 2B). Another example is Glis3, which is a transcription factor and plays a key role in neonatal diabetes, type 1 and type 2 diabetes (Shan et al., 2016). Glis3 produced three circRNA isoforms (circ_0000942, circ_0007671, and circ_0007670) that also share the same acceptor. Another example is Nifa, which co-localizes with Pparγ and transcriptionally induces the brown fat gene expression during brown adipocyte differentiation (Hiraike et al., 2017). As shown in Figure 2C, Nifa produced four circRNA isoforms (circ_Nifa_1, circ_Nifa_2, circ_Nifa_3, and circ_0011879). The alternative splicing was very complex. circNifa1 and circNifa3 shared an acceptor, while circ_0011879 and circNifa2 shared another acceptor. Meanwhile, circ_0011879 and circNifa1 shared a donor.

Next, we explored the expression of these circRNA isoforms. For circRNAs derived from Nedd4, we found circNedd4_1 and circNedd4_2 were the dominant isoforms and the expression patterns of the three circRNA isoforms were completely different. CircNedd4_2 continually increased, while circNedd4_2 decreased on D4. CircNedd4_3 began to increase on D8 (Figure 2D). For the circRNAs derived from Glis3, circ_0000942 increased from D0 to D4, and then start to decrease. Circ_0007671 and circ_0007671 showed similar expression profiles, which kept unchanged until D4 and then decreased to the lowest expression level on D8 (Figure 2E). For the circRNAs derived from Nifa, circ_Nifa_1 and circ_0011879 were the dominant isoforms. Then expression patterns of them were similar, which showed an increase on D4 and then decreased (Figure 2F). In summary, alternative back-splicing expands the diversity of circRNAs, and expression among the back-splicing variants is different.

### Expression Patterns of CircRNAs in BAT Adipogenesis

**FIGURE 3:**
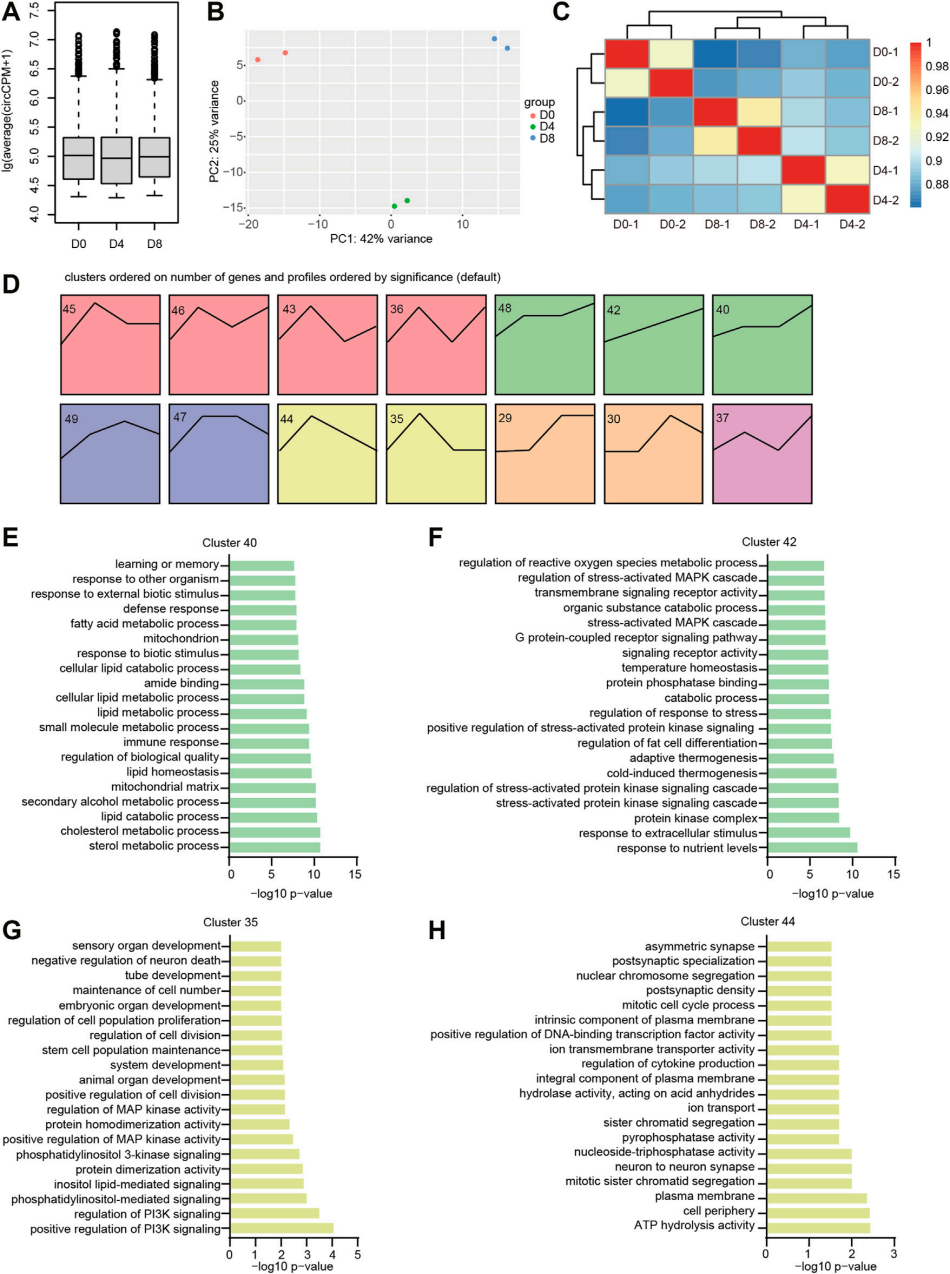
The expression patterns of circRNA during brown adipogenesis. **(A)** Relative expression levels of circRNAs during brown adipogenesis. **(B)** PCA plot of circRNA expression during brown adipogenesis. **(C)** Correlation of circRNA expression calculated by DESeq2 rlog-normalized RNA-seq data. **(D)** Time series analysis of circRNA expression patterns. The number on the top left indicated the expression profile number. **(E–H)** GO terms enriched for the host genes of indicated circRNA clusters.

As mentioned earlier, BAT preadipocytes undergo a tremendous change of morphology during adipogenesis. To examine the overall circRNA expression levels, we did a boxplot and found that the levels of circRNAs during brown adipogenesis were comparable to each other (Figure 3A). To analyze the variation of circRNA expression profiles, we performed PCA. As shown in Figure 3B, the samples derived from the same group were located near each other, indicating the high repeatability of the results. As expected, the samples came from D4 and D8 located nearer than the D0, indicating the circRNA expression patterns were similar in differentiation stages, whereas they were largely different with the proliferation stage. Hierarchical clustering heatmap showed the correlation between different samples ranged from 0.88 to 0.98 (Figure 3C). Replicates in each stage were highly correlated, meanwhile, the D4 and D8 samples were clustered together, which was consistent with the PCA results.

To get an overview of circRNA expression patterns in brown adipogenesis, we carried out a time series analysis by STEM. 14 clusters were significantly enriched (with colored background, Figure 3D), which belong to 6 groups (with the same color). When we examined the GO enrichment results, we found green and yellow clusters were interesting, which correspond to the up and down regulated circRNAs. GO analysis for their parental gene showed the green cluster was associated with brown adipogenesis (Supplementary Table S2). For example, most genes in cluster 40 were associated with lipid metabolisms, such as sterol metabolic process, cholesterol metabolic process, and lipid catabolic process (Figure 3E). The genes in cluster 42 were enriched with GO terms of cold-induced thermogenesis, adaptive thermogenesis, and regulation of fat cell differentiation (Figure 3F). While in the yellow group (clusters 35 and 44), most genes were associated with cell mitosis. For instance, regulation of cell division, mitotic cell cycle process, and mitotic sister chromatid segregation (Figures 3G,H). These results were consistent with the process of brown adipogenesis, in which pre-adipocytes exit the cell cycle, enter the differentiation process, and obtain the function of thermogenesis.

### Differentially Expressed CircRNAs in brown Adipogenesis

**FIGURE 4:**
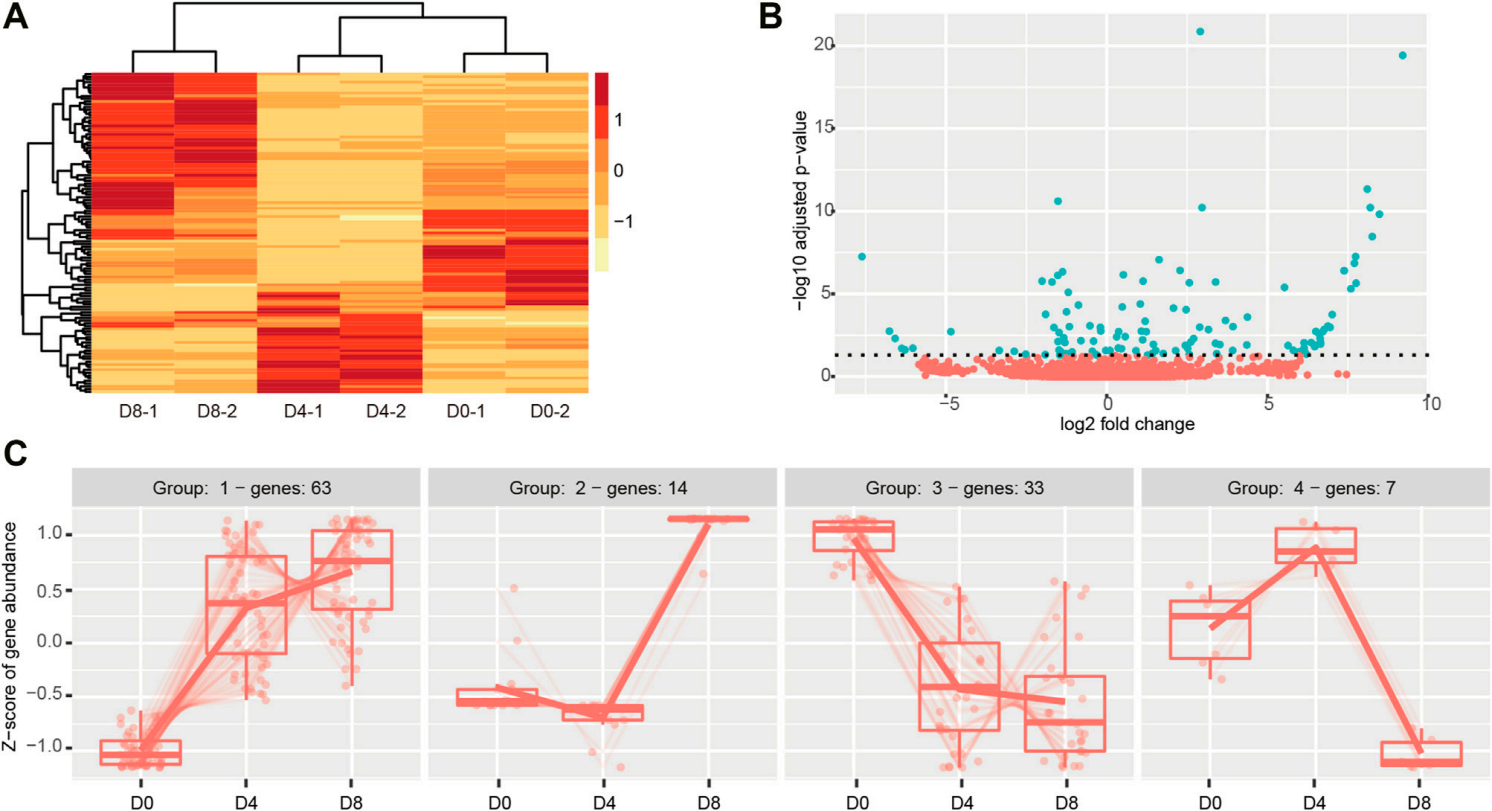
Dynamic differentially expressed circRNAs in brown adipogenesis. **(A)** Heatmap of DECs across different stages of brown adipogenesis. **(B)** Volcano plot showing circRNA abundance across brown adipogenesis. The green color presents the significant changed DECs (padj <0.05). **(C)** Expression patterns of the DECs analyzed by degPatterns function from the DEGreport package (minc = 5).

To examine the DECs in the time-course of brown adipogenesis, we performed the Likelihood ratio test by DESeq2, and the cut-off was set as adjusted *p* < 0.05. As a result, 117 DECs were identified, with 77 upregulated and 40 downregulated (Supplementary Table S3). Notably, much more up-regulated circRNAs were found in the D8 group than in the D0 and D4 groups (Figure 4A). The volcano plot showed log 2 fold change ranged from −7.5 to 10 (Figure 4B). The top upregulated circRNA was circ_Acss3_1, which could not be detected on D0, then it increased to 479 circCPM on D4 and reached an extremely high level on D8 (2428 circCPM).

It has been suggested that circRNA may positively or negatively regulate host gene expression (Shao et al., 2021). To explore the relationship between DECs and their host genes, we collected mRNA expression profiles in brown adipogenesis from our previous study (GEO accession number GSE173710). Then we calculated the correlation of them by Pearson correlation test. We found 40 circRNA-mRNA pairs were significantly correlated (*p* < 0.05, Supplementary Table S4). Interestingly, all of them showed positive correlations, which are consistent with our previous study in white adipogenesis (Zhang et al., 2021a). These results suggested that these DECs may potentially regulate their host genes expression in brown adipogenesis.

It is reported that some of the circRNAs showed highly evolutionary conservation between humans and mice (Jeck et al., 2013). To evaluate the conserved circRNAs, we obtained the human circRNA sequences from circBase (Glažar et al., 2014). Then, alignments were conducted using the NCBI-BLAST-2.11.0+ (https://www.ncbi.nlm.nih.gov/books/NBK131777/) to identify the regions of the mouse circRNAs that corresponded to the human circRNAs. Among the 117 mouse DECs, 85 of them aligned to human circRNAs (E value <10^−5^, Supplementary Table S5). In addition, it should be noted that the number of the conserved circRNAs may be underestimated, as circRNAs are tissue-specific and their expression profile in human BAT is not reported yet. These results indicated that a high ratio of the DECs is conserved between mice and humans.

According to the previous study, the circSAMD4A (derived from host gene SAMD4A, also known as SAMD4) regulated white pre-adipocytes differentiation (Liu et al., 2020). In the current study, we also found the circRNA was differentially expressed in brown adipogenesis, suggesting that it may also be involved in brown adipogenesis. Another reported circRNA derived from Arhgap5 (Arcinas et al., 2019) was also detected in our study, but it was not significantly differentially expressed in brown adipogenesis.

Then, we classified the genes that exhibited similar change across adipogenesis by using DEGreport which uses a hierarchical clustering approach based on pair-wise correlations. The DECs were classified into four groups (Figure 4C; Supplementary Table S3). The genes included in each group ranged from 7 to 63. The largest one was group1, which contained 63 circRNAs with a continuously increasing trend during adipogenesis. Then, it was group 3, which contained 33 circRNAs and kept on decreasing in adipogenesis. Group 2 contained 14 circRNAs which showed a sharp increase after D4. Group 4 contained only 7 circRNAs which were found to increase on D4 and then declined on D8. Although GO analysis showed no terms were significantly enriched, we identified many DECs parental genes which are essential in adipogenesis and brown adipocyte bioenergetics, such as Pparγ, Zbtb16, Sik2, Snrk, Nfia, and Tead1.

### Validation of circRNAs

**FIGURE 5:**
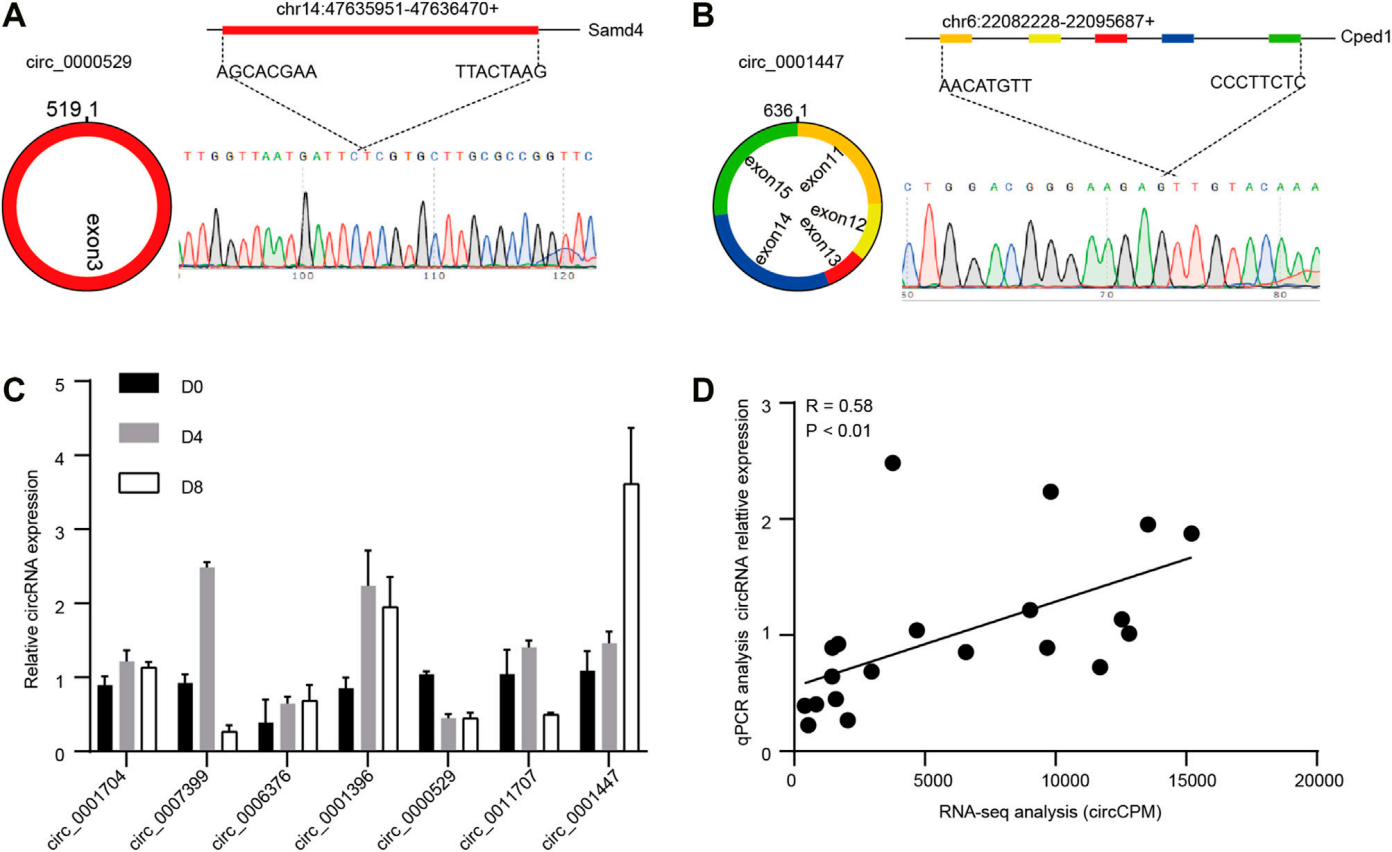
Verification of circRNAs. Representative circRNA generation models and Sanger sequencing results of circRNAs **(A)** circ_0000529, **(B)** circ_0001447. **(C)** Relative circRNA expression levels were detected by qPCR. (D) Correlation analysis of RNA-seq results and qPCR results (*n* = 3).

To determine the authenticity of the RNA-seq results, seven of the DECs were selected randomly to detect the back-splicing sites by Sanger sequencing. The circRNA sequence and genome locus were obtained by circPrimer (version 2.0). Then, we performed RT-PCR using divergent primers (version 4.1.0, Supplementary Table S6) and all the circRNAs were successfully amplified. We collected the corresponding DNA products and performed Sanger sequencing, as shown in Figures 5A,B, the expected back-splicing sites were detected. Then, the relative circRNAs expression levels were detected by qPCR. Compared to RNA-seq results, the expression trend of most circRNAs was similar. (Figure 5C). Further, we found the qPCR and RNA-seq results were significantly correlated (R = 0.580, *p* < 0.01, Figure 5D).

### Prediction of microRNA-circRNA Interaction

**FIGURE 6:**
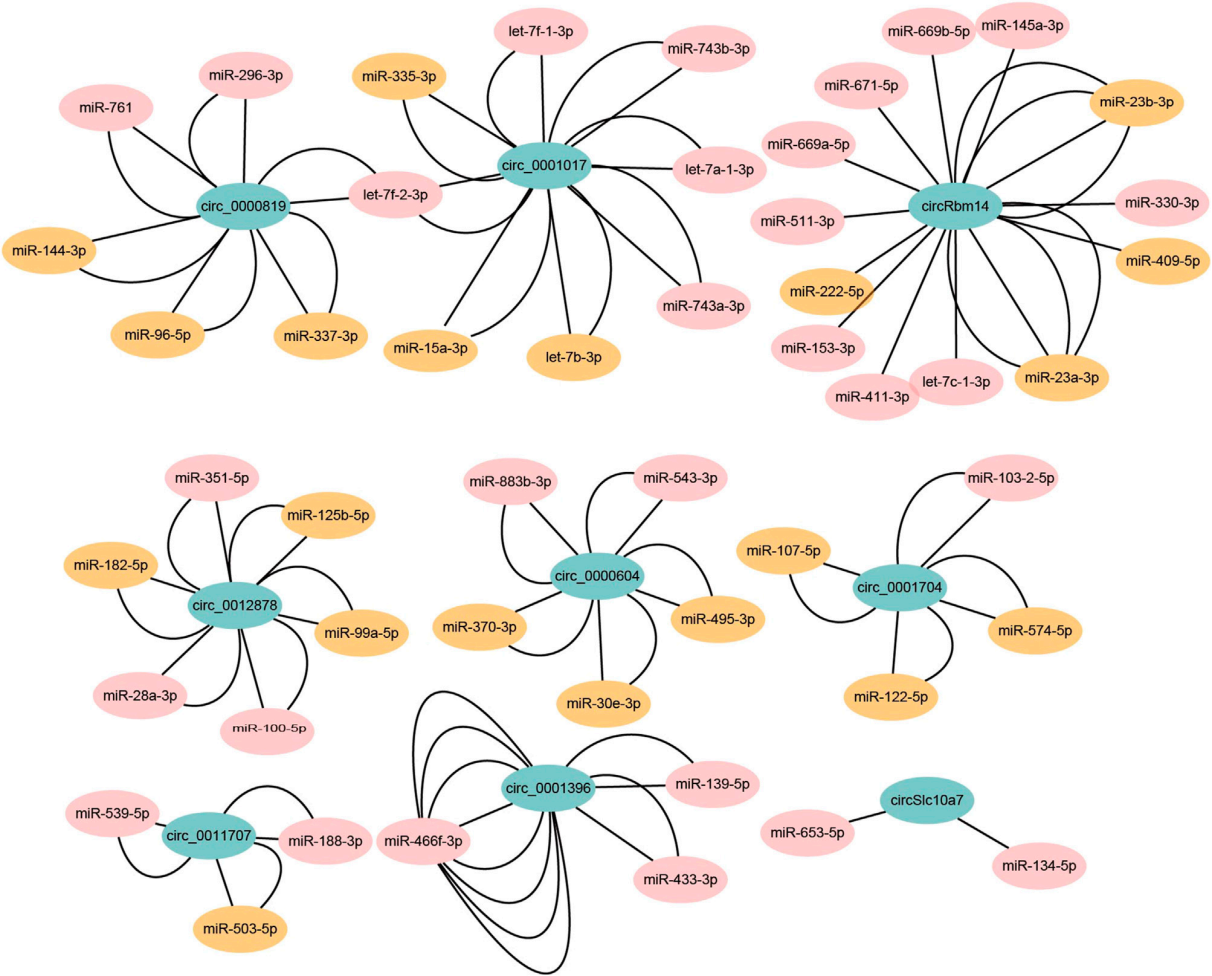
The predicted circRNA-miRNA interaction networks. The potential miRNA binding sites of circRNAs were predicted by miRanda. The edge number represents the number of miRNA binding sites in the indicated circRNA sequences. The miRNAs with brown color indicate that these miRNAs are associated with adipogenesis or metabolic diseases according to HMDD (version 3.2) or previous study.

Recently, it has been reported that circRNAs can act as miRNA sponges and repress miRNA functions (Hansen et al., 2013). As miRNAs control a large set of biological processes in adipogenesis, circRNAs may be involved in the process through miRNAs (Zhong et al., 2019). To validate the hypothesis, we chose the top 10 abundant DECs to predict the potential circRNA-miRNA interactions with miRanda. We found all of them were predicted to contain miRNA binding sites, except circ_0001511. To reduce false positives, we only kept the miRNAs that could be detected in brown adipogenesis (GEO accession number GSE45499) (Chen et al., 2013). In the end, we predicted 49 miRNAs may interact with the DECs. Cytoscape was used to construct the miRNA-circRNA interaction networks (Figure 6; Supplementary Table S7). Notably, each circRNA contained several different miRNA binding sites and a total of 97 miRNA binding sites were identified. More interestingly, most of the circRNAs contained multiple binding sites for the same miRNA, for example, circ_001396 was predicted to contain as many as 8 binding sites for mir-466f-3p, which greatly increased the possibility of their interaction.

The Human microRNA Disease Database (HMDD) collects experiment-supported miRNAs associated with disease (Huang et al., 2019). We further explored the potential biological functions of the identified miRNAs. According to HMDD (version 3.2) and published data, 20 of the 49 miRNAs were revealed to be associated with adipogenesis or metabolic diseases, including obesity, mitochondrial metabolism disease, type 2 diabetes, and non-alcoholic fatty liver disease (Figure 6; Supplementary Table S8). For example, let-7, miR-337, miR-503, and miR-182 were identified in the networks. They were reported to play regulatory roles in adipogenesis or thermogenesis. In addition, miR-15a and miR-30e may be involved in mitochondrial metabolism disease and obesity, respectively. In summary, the DECs may potentially interact with these miRNAs to exert their regulatory functions.

## Discussion

Previous studies had shown that a lot of circRNAs were expressed in adipose tissue. During Yak adipocytes differentiation, 7,203 circRNA were detected (Zhang et al., 2020). In human WAT, 6,925 circRNAs were detected, while in mouse WAT 2,380 circRNAs were detected (Arcinas et al., 2019). We previously showed 3,771 circRNA were detected during mouse white adipogenesis. However, the circRNA expression in BAT has not been reported. In the present work, the expression profiles of circRNAs were investigated during brown adipogenesis. As many as 3,671circRNAs were identified and 36.9% of them were not annotated yet. This is the first study that reports the expression of circRNAs expression profiles in brown adipogenesis. Compare to our previous work studying circRNA in white adipogenesis, we found 1,144 of the circRNAs were specifically expressed in BAT. Further, we showed many circRNA were differentialy expressed during brown adipogenesis. These results are consistent with the idea that circRNAs are tissue-specific and developmental stage specific.

The functions of many circRNAs remain unclear at present. However, accumulated reports suggest they can regulate parental gene expression through various mechanisms (Shao et al., 2021). As both circRNAs and linear RNAs are generated from pre-RNA, they compete with each other for splicing, thus decrease the expression levels of parental genes (Ashwal-Fluss et al., 2014). On the other hand, circRNAs can increase parental gene expression. For example, circEIF3J and circPAIP2 bind to RNA Pol II and U1 snRNA, then activate parental genes transcription. Circ-Sirt1 can compete with miR-132/212 to bind Sirt1, leading to the enhancement of Sirt1 expression (Kong et al., 2019). CircRNA sisR-4 promoted parental gene expression by activating enhancer (Tay and Pek, 2017). Based on the idea that circRNAs may regulate parental genes expression, we predicted their potential functions. According to the annotation of the parental genes, downregulated circRNAs were enriched in GO terms related to cell proliferation and cell cycle, whereas the up-regulated circRNAs were enriched in cell differentiation and thermogenesis. These results were consistent with the activity of brown adipogenesis. At the early stage of brown adipogenesis, preadipocytes undergo a post confluent mitosis and exit the cell cycle. Then the pre-adipocytes are committed to adipocytes. A batch of adipose-related genes began to accumulate (Ntambi and Young-Cheul, 2000). In addition, we found many parental genes of the DECs participated in adipogenesis modulation. For example, Pparγ is indispensable for adipogenesis (Brun et al., 1996), which produced five circRNA isoforms and one of them showed a continuously increasing expression. Circ_0001335 derived from Nsd2 which inhibits H3K27me3 and increases expression of C/EBPα and Pparγ, thus promoting adipogenesis (Brun et al., 1996). Fkbp5 and Fndc3b are essential for adipogenesis (Muraoka et al., 2009; Li et al., 2018b), both can also produce circRNAs. Meanwhile, some of the DECs parental genes are involved in regulating brown adipocyte bioenergetics. Parental gene Zbtb16 is a transcription factor that is induced upon cold exposure. It can increase the expression of BAT marker genes and β-oxidation genes (Plaisier et al., 2012). Another circRNA parental gene was Sik2. Sik2/TORC2 signaling cascade regulates PGC-1α and UCP-1 gene expression in BAT (Muraoka et al., 2009). In addition, several other circRNA parental genes, such as Snrk (Li et al., 2018b), Nfia (Hiraike et al., 2017), and Tead1 (Tharp et al., 2018) are also involved in BAT-specific genes expression and bioenergetics. In summary, we find many parental genes are key adipogenesis regulators.

It is reported that circRNA can post-transcriptional modulate gene expression by binding miRNA. Several circRNAs have been reported to affect adipogenesis. In obese individuals, CircSAMD4A expression increased. It could bind with miR-138-5p and induce adipogenesis (Yang et al., 2011; Liu et al., 2020). In bone marrow mesenchymal stem cells, CDR1as increases adipogenesis by binding with miR-7-5p (Chen et al., 2020). In the current study, we identified that 9 of the top 10 abundant DCEs contained miRNA binding sites. We found circ_0001017 was predicted to contain several Let-7 binding sites. Let-7 is one of the well-studied miRNAs which inhibits pre-adipocytes clonal expansion and terminal differentiation via targeting HMGA2 (Sun et al., 2009). MiR-337-3p can inhibit TWIST1 and promote transcription of genes participating in brown fat metabolism (Vonhögen et al., 2020). In circ_0000819, two miR-337-3p binding sites were identified. In addition, we found that circ_0001017 may interact with miR-503 which was reported to regulate brown adipogenesis by inhibiting BMPR1a (Man et al., 2020). Thus, these DECs may be involved in brown adipogenesis through post-transcriptional regulation by binding with miRNAs.

## Conclusion

Collectively, we characterized circRNA expression patterns during brown adipogenesis. We identified a large number of novel circRNAs and found alternative back splicing is an important source of circRNA diversity. We found many of the circRNAs are brown adipogenesis and differentiation stage specific. We also predicted that adipose circRNAs may regulate adipogenesis by acting as microRNA sponges. These novel circRNAs may potentially serve as new regulators of BAT development.

## Data Availability

The datasets presented in this study can be found in online repositories. The names of the repository/repositories and accession number(s) can be found in the article/Supplementary Material.
